# An Improved Test Method for Assaying the Inhibition of Bioflavonoids on Xanthine Oxidase Activity *in vitro*

**DOI:** 10.1002/open.202400127

**Published:** 2024-09-09

**Authors:** Yuanyong Yao, Tao Wu, Meng Zhang, Daihua Fu, Hai Yang, Shixue Chen

**Affiliations:** ^1^ State Ethnic Affairs Commission Key Development Laboratory of Chinese Veterinary Medicine & National and Local Joint Engineering Center of Chinese Veterinary Medicine Separation and Purification Technology Tongren Vocational and Technical University Tongren 554300 China; ^2^ Institute of Material and Chemical Engineering Tongren University Tongren 554300 China; ^3^ Key Laboratory of Medicinal Chemistry for Natural Resource of Ministry of Education Yunnan University Kunming 650091 China

**Keywords:** Test method, Xanthine oxidase, Bioflavonoids, In vitro inhibition

## Abstract

The difference on inhibitory effects of bioflavonoids inhibiting XOD activity assayed by varying test methods cause of us to be further in consideration. The reported test method creating a micro‐environment surrounding XOD in the absence of ⋅O_2_^−^, which is seemly different from the assay *in vivo*. So, the *vitro* test method for assaying XOD activity is necessary to be improved for selection of potential inhibitors in the presence of ⋅O_2_^−^. The inhibitory results demonstrated that bioflavonoids of MY, DMY, QUE and LUT are capable to be on effective IC_50_ values, but others are not. As well, their resulting inhibitions determined by the improved test method are much less than that reported in the literature, indicating that their chemical affinities with XOD become weaker. Moreover, DMY assayed on the inhibitions of XOD in the improved test method performs to be a better inhibitor, as compared to the assay of the reported test methods. Abasing on the transformation of DMY into MY in the presence of ⋅O_2_^−^, the good inhibition of DMY on XOD activity can be explained by the synergistic effect of MY.

## Graphical abstract

The test method for assaying inhibitory effects of bioflavonoids on xanhine oxidase *in vitro* has been improved successfully with the equipment of UV‐Vis technique. In the improved test method, there is a micro‐environment surrounding XOD has been created in the presence of •O_2_^‐^, which is similar to that of *in vivo*. Simultaneously, the inhibition of dihydromyricetin as a chiral flavonoid was effectively assayed by the improved test method, indicating dihydromyricetin enabling to be a potent inhibitor of XOD, resulting from the selective transformation of DMY to MY occurred.

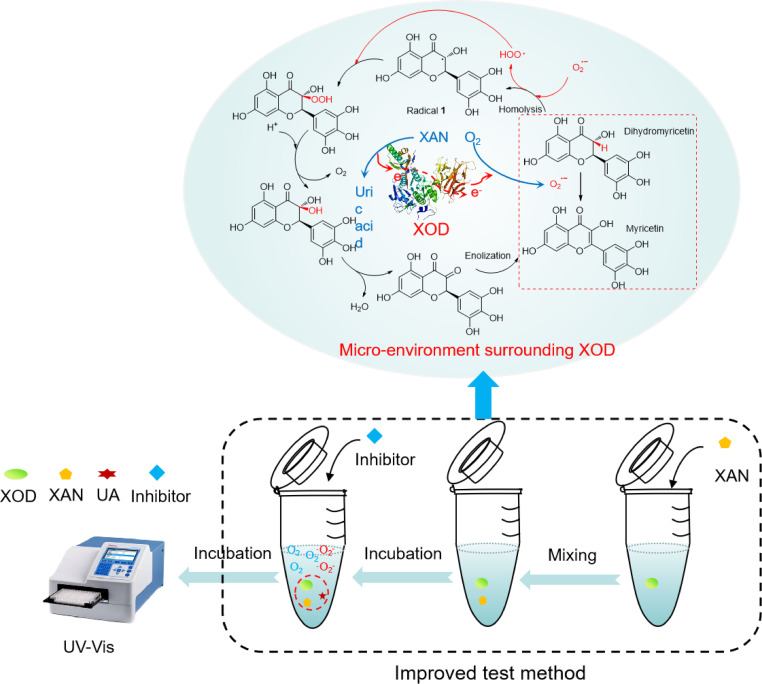

## Introduction

**Figure 1:**
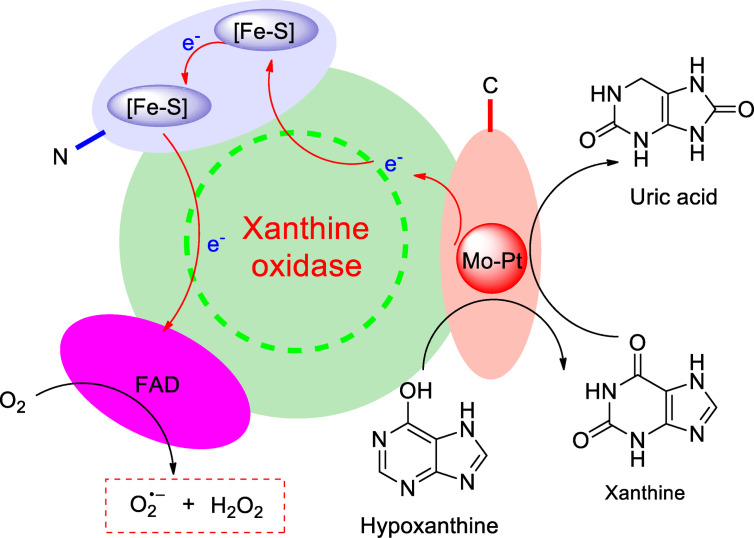
Catalytic oxidation of xanthine into uric acid promoted by XOD.

A better understanding of the role that enzymes play in the basic life support of living organisms is of considerable interest. Xanthine oxidase (XOD) acting as a ubiquitous metallo‐flavo enzyme exists widely in the various tissues of mammals, performing a crucial role in physiological and pathological processes. The enzyme is a homodimer of 290 kDa, with two identical subunits. Each subunit contains N‐terminal domain binding to two Fe_2_S_2_ type iron‐sulfur clusters, a flavin adenine dinucleotide (FAD) cofactor and a C‐central molybdopterin center (Figure 1).^[1]^ Relying on the particular framework of subunit, the oxidation of hypoxanthine to xanthine or xanthine to uric acid can be efficiently catalyzed in purine catabolism, as well as generation of superoxide anion radical (⋅O_2_^−^), which is a great significance in metabolism of mammals.^[2]^ Uric acid acting as a terminal oxidative product of xanthine is vulnerable to bring about the hyperuricemia, when overproduced or underexcreted in bloods of mammals.^[3]^ Large amounts of uric acids are deposited on the joint or tissues by crystal or its salt through blood circulation, it is capable to cause of gout.^[3]^ Hence, XOD has been certainly used as an effective target in the clinic treatment of hyperuricemia. Its activity is highly deserved to be concentrated.

At the present, the approach to assaying biological enzymatic inhibition *in vitro* is the measurement of the quantities of generating product or decreasing catalytic substrate in the presence of inhibitor. XOD is an oxidized enzyme, can efficiently oxidize xanthine into uric acid in the kinetic enzymatic model. In general, identifying amount of uric acid generating from xanthine promoted by XOD has been popular to be a crucial index for evaluating inhibitory effect of inhibitor *in vitro*, because of uric acid as a catalytic product enabling to perform the characteristic absorbance occurred at 292±2 nm, which can be obviously observed in UV spectrum, with a good concentration‐dependence.^[4]^ So, in term of assaying XOD inhibition, UV‐Vis technique has been widely utilized to assaying XOD inhibition *in vitro*. As far as we known, the reported test method equipped with UV technique to assaying XOD inhibition *in vitro* has been widely applied to the selection of potent potential inhibitors.[^5^, ^6^, ^7^] Furthermore, the reported test method is usually asked for inhibitor to mix with XOD to form the complex, before the catalytic oxidation initiated by the addition of xanthine as a substrate.[^8^, ^9^, ^10^, ^11^, ^12^, ^13^, ^14^] In recent years, there are some natural compounds can be witnessed to be good potential inhibitors of XOD *in vitro* by the reported test method. For instances, myricetin as a plant‐derived flavonol, isolated from Cortex Myricae, was found to be good effect in the inhibition of XOD, with an IC_50_ value of 8.66±0.03 μmol L^−1^.^[8]^ Quercetin was reported to be 2.74±0.04 μmol L^−1^ at IC_50_ value, presenting good chemical affinity with XOD *in vitro* occurred.^[9]^ Hesperetin,^[10]^ baicalein,^[11]^ pinobanksin,^[12]^ galangin,^[12]^ chrysin^[13]^ and fisetin^[14]^ acting as popular bioflavonoids were respectively investigated to be potential inhibitors of XOD with IC_50_ values of 50.30, 2.79, 137.00, 163.00, 5.02 and 4.33 μmol L^−1^.

Moreover, it is worth noting that the micro‐environment surrounding XOD created by the reported test method *in vitro* is absolutely distinguished from that of *in vivo*. Because XOD oxidizing xanthine is a dynamic process *in vivo*, with continuously generating ⋅O_2_^−^. Inhibitor is planing to make an interaction with XOD *in vivo*, it has to tolerate the attack of ⋅O_2_^−^generating from XOD. ⋅O_2_^−^ is a active oxygen species, performing strong redox capacity,^[15]^ it is flexible for the molecular skeleton of inhibitor to be affected to influence its inhibitory effect on XOD activity, especially for bioflavonoids acting as potential inhibitors. Therefore,the micro‐environment surrounding XOD created is predominant in the assay of bioflavonoids on the inhibition of XOD activity *in vitro*.

Herein, the aim of this work is to create a micro‐environment surrounding XOD *in vitro* similar to that of XOD *in vivo*, by which the test method to assaying XOD inhibition *in vitro* is improved. Subsequently, there are some bioflavonoids to be selected for investigation on the inhibition of XOD activities by the improved test method. As well, their inhibitory results are required for a comparison with those of identical bioflavonoids in the reports. Eventually, effective bioflavonoids are selected to be respectively investigated on the inhibition of XOD by the reported and improved test method, so that it indicates that the improved test method to assaying XOD inhibition *in vitro* is a potent gateway to select potential inhibitors of XOD.

## Experimental Section

### Chemicals

Xanthine oxidase (approximately 7.8 U mg^−1^) and xanthine (XAN) were purchased from Yuanye Biotechnology Co. (Shanghai, China). The stock solution of XOD (0.0010 g mL^−1^) was prepared in 0.1 mol L^−1^ sodium pyrophosphate buffer (pH=7.2/7.5/7.8/8.1/8.5), which was accurately weighing sodium pyrophosphate (5.3180 g) to be dissolved into distilled water (200 mL) with EDTA (0.0175 g), and then adjusted to be 7.2/7.5/7.8/8.1/8.5 in pH value by phosphoric acid. Varied concentrations of XOD solution and 1 mmol/L of XAN solution were respectively diluted by the stock solution of XOD (0.0010 g mL^−1^) and XAN (50 mmol L^−1^), using sodium pyrophosphate buffer (pH=7.2/7.5/7.8/8.1/8.5). ALL of testing samples including bioflavonoids and allopurinol (ALL) were obtained from Aladdin Industrial Co. (Shanghai,China), except for dihydromyricetin (DMY) isolated from tender stem and leaves of Ampelsis grossedentata in greater 98 % purity in our laboratory. They were dissolved in distilled water with little DMSO as stock solutions (750 μmol L^−1^). And these stock solutions were authorized to be diluted with distilled water to be different concentrations as required in experimental process. Standard substance of uric acid was purchased from Aladdin Industrial Co. (Shanghai, China). Nitroblue tetrazolium (NBT) were purchased from Yuanye Biotechnology Co. (Shanghai, China). ALL of solutions asked for immediate use, and all other reagents and solvents were of analytical reagent grade.

### Instrumentation

UV spectra were recorded at room temperature, using an UV‐Vis spectrophotometer (UV‐759S) equipped with the wavelength in the range of 200 nm to 800 nm (Light Technology Co, Shanghai, China).

### Constructing XOD‐XAN Dynamic Model in vitro

#### Preparation of Uric Acid Working Curve

The standard substance of uric acid was accurately weighed to be 0.0025 g and dissolved into sodium pyrophosphate buffer to be varying concentrations of 2.5, 5.0, 7.5, 10.0, 12.5, 17.5, 22.5, 32.5 and 42.5 μg mL^−1^. Each concentration of uric acid solutions were respectively asked to be scanned by UV‐759S spectrophotometer equipped with a 1.0 cm path length cell. The absorbance at 294±2 nm was required to be recorded for building up the relationship of between uric acid concentrations and their absorbance.

#### General Procedure for Constructing XOD‐XAN Dynamic Model

The product of uric acid generated from the catalytic oxidation of XAN by XOD was selected to be an essential index, by which the XOD‐XAN dynamic model *in vitro* was constructed. The general procedure for constructing XOD‐XAN dynamic model *in vitro* should be as follows: a 1.6 mL of XOD solution at a fixed concentration was allowed to be mixed completely with xanthine solution (1 mmol L^−1^, 1.0 mL) and 0.4 mL sodium pyrophosphate buffer. Then the reacting mixture was incubated for several minutes at different temperature. Afterwards, the resulting mixture was authorized to be scanned spectrophotometrically at the wavelength of 294±2 nm to identify crucial factors affecting XOD bio‐activity *in vitro*.

### Assessment of Factors Affecting the Enzymatic Activity of XOD in vitro

#### Incubation Temperature

A series of 3.0 mL reaction mixture consisting of a fixed concentration of XOD (1.6 mL, 0.0128 mg mL^−1^), sodium pyrophosphate buffer (0.4 mL, pH=7.5) and xanthine solution (1.0 mL, 1 mmol L^−1^) were prepared and incubated for 15 min at 25, 37 and 45 °C, respectively. Then these reacting mixtures were subjected to the UV‐759S spectrophotometer for observing the amount of uric acid produced in XOD‐XAN dynamic model *in vitro*, by measuring the absorbance at 294±2 nm.

#### The Value of pH

Buffer solutions at pH=7.2, 7.5, 7.8, 8.1 and 8.5 were prepared by dissolving sodium pyrophosphate/phosphoric acid in distilled water. XAN as catalytic substrate and XOD were respectively asked to be dissolved completely into the sodium pyrophosphate buffer to be a fixed concentration. Then the oxidation of XAN *in vitro* was allowed to proceed for 15 min at 37 °C. Under observation of UV‐759S spectrophotometer with the wavelength of 294±2 nm, their absorbance was measured and recorded to select appropriate pH surroundings of XOD *in vitro*.

#### Incubation Time

A mixture consisting of sodium pyrophosphate buffer (0.4 mL, pH=7.5) and XOD (1.6 mL, 0.0128 mg mL^−1^) was prepared and incubated for 5 min at 37 °C. Then the reaction was initiated by adding substrate XAN (1.0 mL, 1 mmol L^−1^) and permitted to run for 1, 3, 6, 9, 12, 15, 18, 21 and 24 min respectively under the same conditions. With the measurement of absorbance at 294±2 nm, the factor of incubation time was optimized for constructing XOD‐XAN dynamic model *in vitro*.

#### Quantity of XOD

The stock solution of XOD (0.0010 g mL^−1^) was diluted to be respective 0.0004, 0.0007, 0.0013, 0.0038 and 0.0128 mg mL^−1^ in concentrations with sodium pyrophosphate buffer (pH=7.5). Each concentration of XOD solution (1.6 mL) was respectively mixed with xanthine solution (1 mmol L^−1^, 1.0 mL) and 0.4 mL sodium pyrophosphate buffer. The resulting mixture was permitted to incubate for several minutes at 37 °C, the appropriated quantity of XOD was assayed by measuring the quantity of uric acid in production.

### Assay of ⋅O_2_^−^ Produced by XOD‐XAN Dynamic Model

The production of ⋅O_2_^−^ was determined by the reduction of NBT into a blue formazan, which was absorbed at 560 nm.^[16]^ The test solution was a reacting mixture consisting of XOD (1.6 mL, 0.0128 mg mL^−1^), XAN (1 mmol L^−1^, 1.0 mL), sodium pyrophosphate buffer (0.38 mL, pH=7.5) and NBT (0.02 mL, 2.5 mmol L^−1^). The mixture was permitted to be incubated for 5–50 min at room temperature. And then the absorbance of the mixture was recorded at 560 nm.

### Evaluation on XOD Activity in vitro

Under optimized conditions, a reaction mixture composed of a fixed amount of XOD (1.6 mL, 0.0128 mg mL^−1^) and xanthine (1 mmol L^−1^, 1.0 mL) was prepared and incubated for 5 min at 37 °C. And then potential inhibitor(s) (0.4 mL) at different concentrations was allowed to be added. The reaction was continuously incubated for 10 min under the same conditions. Subsequently, the resulting mixture was subjected to UV spectrophotometer for scanning at 294±2 nm. The absorbance was recorded to be a ΔA1 .

The reaction mixture of XOD and XAN incubated for 5 min at 37 °C was regarded as a background, with the absorbance at 294±2 nm recorded to be a ΔA2 . The symbol of ΔA0 represented the absorbance obtained from the catalytic oxidation of XAN by XOD for 15 min at 37 °C. Allopurinol was used as a positive control. The relative enzymatic activity was assayed by the measurement of uric acid formation *in vitro*. The inhibitory effect of XOD activity *in vitro* was depicted by the following equation:

$$I\;\%=\frac{\;\Delta A_{0\;}-\Delta A_{1}}{\Delta A_{0\;}-\Delta A_{2}}\times 100\;\%$$

## Results and Discussions

### Establishment for Micro‐Environment Surrounding XOD

**Figure 2:**
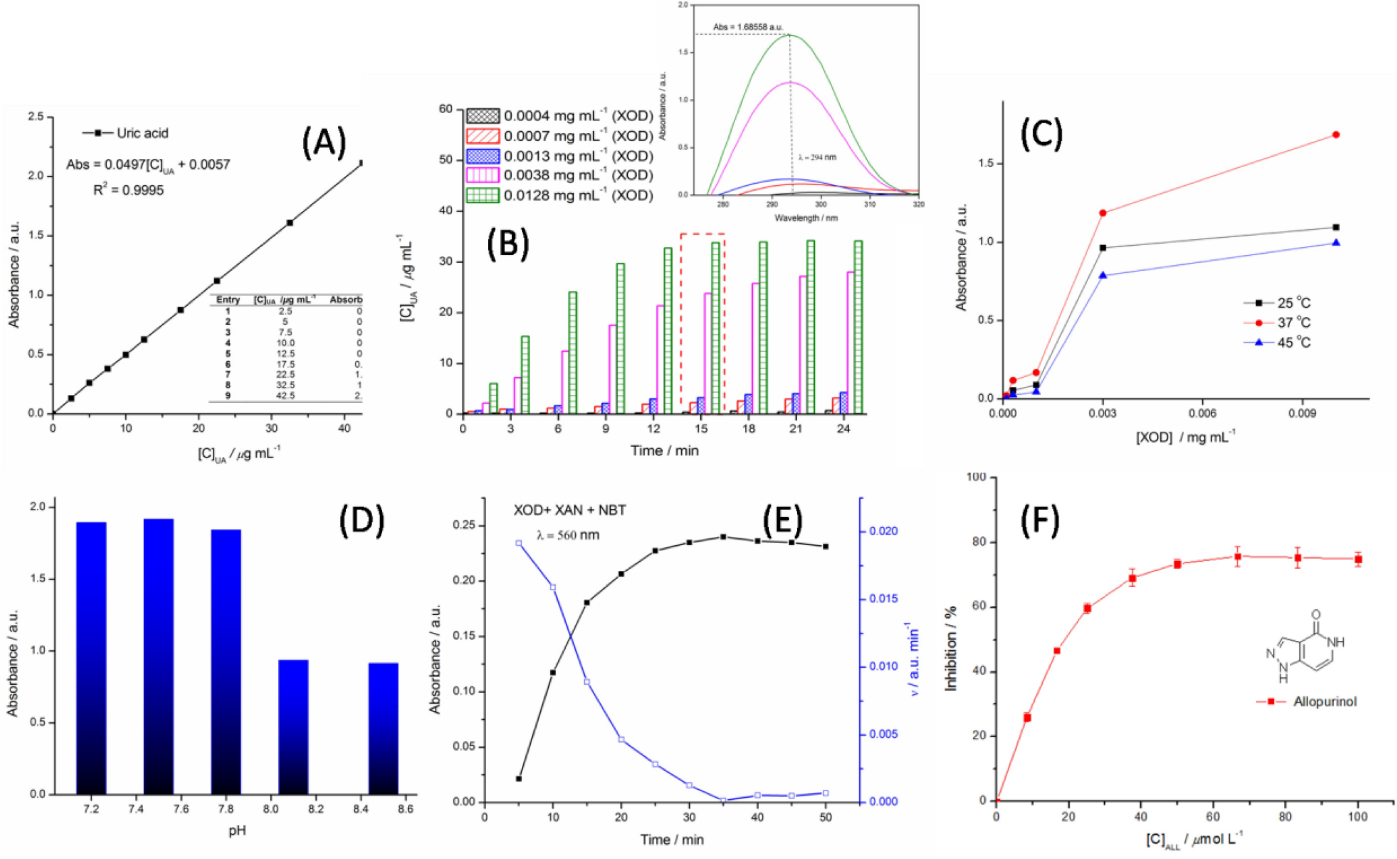
Optimization for *in vitro* XOD‐XAN dynamic model by factors.

To the best of our knowledge, creating an enzymatic micro‐environment is essential to develop an *in vitro* enzyme dynamic model. The *in vitro* dynamic model of catalyzing oxidation of xanthine into uric acid is optimized by investigating varying concentrations of XOD, pH value, incubation time and temperature as factors. The inhibition of XOD is evaluated by the measurement of amount of uric acid generating from the oxidation of xanthine as a substrate under certain conditions. The values of uric acid at concentrations are determined by the working curve of standard uric acid and the absorbance occurred at 294±2 nm (Figure 2 (A)). ⋅O_2_^−^ generated by XOD is detected by the reduction of NBT into a blue formazan, whose characteristic absorbance is at 560 nm.^[16]^ In this work, the trial is aimed to hunt for reaction termination of enzymatic oxidation and identify some factors affecting enzymatic biological activities for creating an optimum micro‐environment surrounding XOD *in vitro*. Firstly, vital factors of both enzymatic concentrations and incubation time affecting the construction of the XOD‐XAN dynamic model *in vitro* were observed by UV technology. As shown in Figure 2 (B), with the increase of concentrations of XOD solution from 0.0004 to 0.0128 mg mL^−1^, it was obviously seen that the absorbance at 294±2 nm perform to be enhancing under the same conditions. That is to say, the contents of XOD in aqueous solution are directly proportional to the production of uric acid in quantity, with xanthine as a substrate incubating for 15 min at 37 °C. Furthermore, when the concentration of XOD was fixed *in vitro* test, the factor of incubation time affecting enzymatic bio‐activity was affirmed to be an important building block, via observing the increase of uric acid in amount, as incubation time prolonging at certain range. As well, temperature affecting the *in vitro* XOD‐XAN dynamic model should be attributed to the influence on biological activity of XOD, because of enzymatic conformation being sensitive to thermal surroundings. In Figure 2(C), it was well indicated that the temperature of 37 °C exhibits an appropriate thermal surroundings for the XOD‐XAN dynamic model *in vitro*, with the higher absorbance occurred at 294±2 nm, as compared to thermal surroundings of 25 °C and 45 °C. Simultaneously, the value of pH as another factor was not ought to be ignored in optimizing construction conditions of the *in vitro* XOD‐XAN dynamic model, because the catalytic capacity of XOD at the active site was closely related to the surroundings of pH. As depicted in Figure 2(D), when pH value of XOD surroundings was adjusted from 7.2 to 7.8, the change of the absorbance occurred at 294±2 nm was not obviously observed, but their absorbance enabled to reach up to 1.68558 a.u. in value, indicating the production of uric acid with 33.80 *μ*g mL^−1^ in concentration. Moreover, with the increase of pH value from 7.8 to 8.5, it was well distinguished that the absorbance at 294±2 nm exhibits an apparent decline in value, implying the production of uric acid being less in amount. So the value of 7.5 in pH was affirmative to be appropriate XOD surroundings. Secondly, under above optimum conditions, the quantity of ⋅O_2_^−^ generated by XOD performed to be directly proportional to incubation time at certain range. The *in vitro* XOD‐XAN dynamic model was incubated for 5 min, the rate of producing ⋅O_2_^−^ was relative maximum, which was shown in Figure 2(E). As prolonging incubating time, a decline on the rate of generating ⋅O_2_^−^was obviously appeared up. Eventually, through the optimization for the *in vitro* XOD‐XAN dynamic model, the micro‐environment surrounding XOD has been successfully established with the generation of ⋅O_2_^−^ *in vitro*.The improved test method to assaying XOD inhibition with a micro‐environment surrounding XOD similar to that of XOD *in vivo* can supply a support for potential biofalvonoids on the inhibition of XOD.

### Assaying XOD Inhibition in vitro

**Figure 3:**
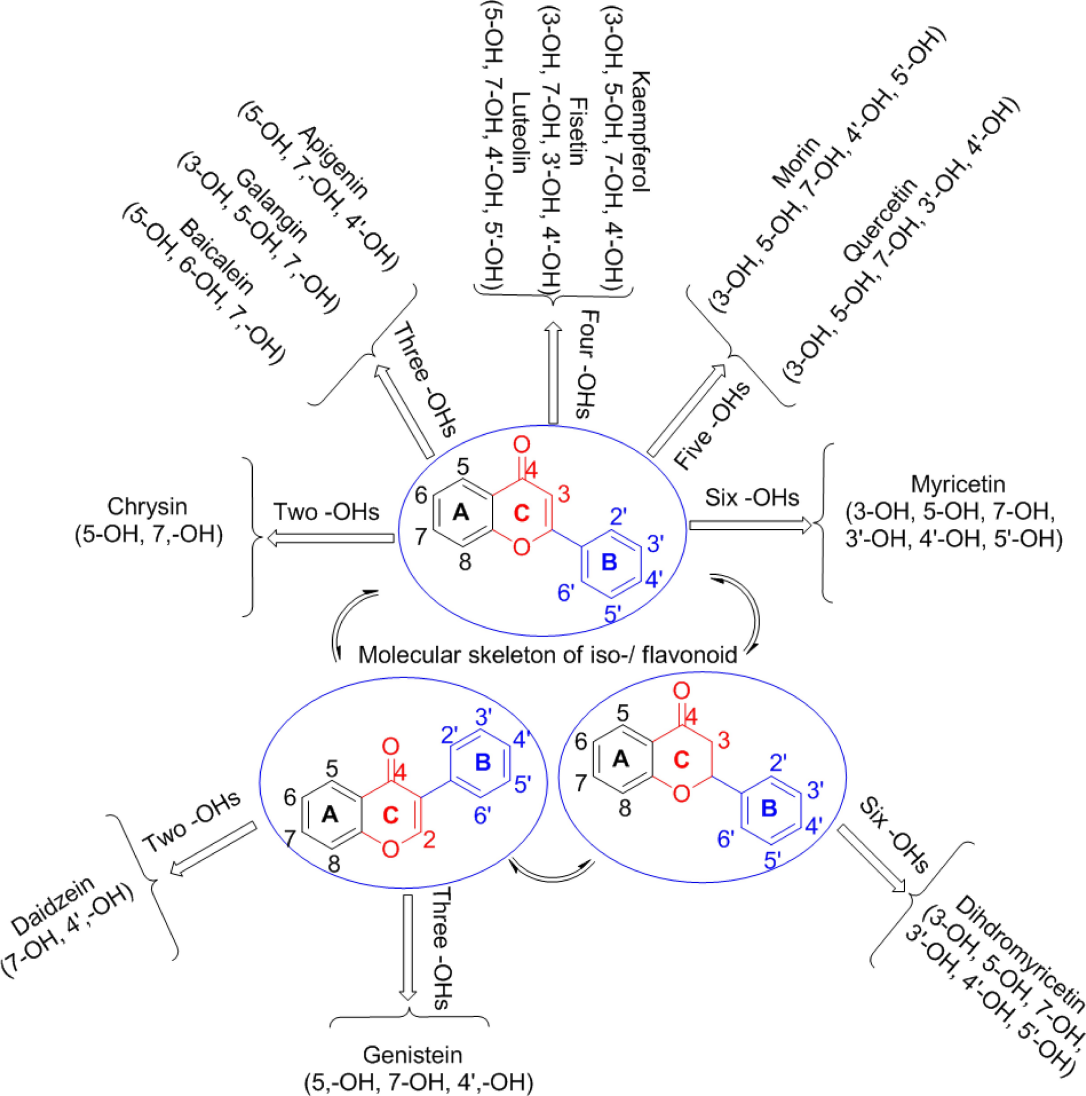
Molecular skeletons of bioflaonoids with the attachment of hydroxyl moieties.

**Figure 4:**
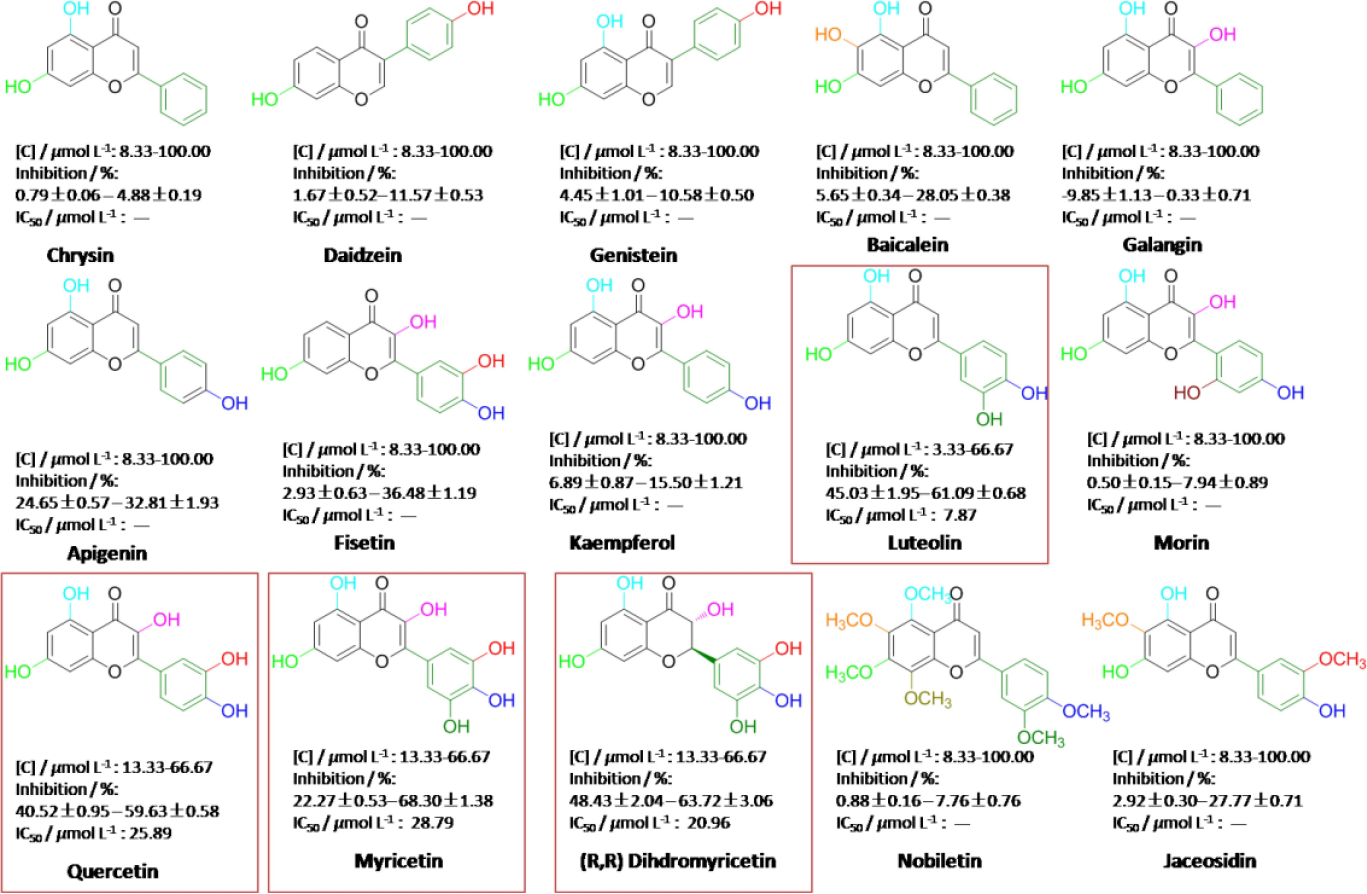
Inhibitory effects of bioflavonoids on XOD activity *in vitro*.

Next, a clinic inhibitor of ALL acting as a potent agent was employed to evaluating the inhibition of XOD by the improved test method. As shown in Figure 2 **(F)**, the inhibitory results demonstrated that the inhibition of XOD *in vitro* is closely correlated to dosages of ALL, presenting good stability and availability. Afterwards, some of bioflavonoids were systematically investigated on the inhibition of XOD by the improved test method, which were shown in Figure 3. Chrysin and daidzein with two hydroxyl moieties distributed respectively on 5, 7‐ and 7, 4’‐ positions of molecular skeleton, are named as a flavone and an isoflavone. In the assay of *in vitro* XOD inhibition, the inhibitory effect of daidzein exhibits a better performance than that of chrysin under the same conditions, they are respective 11.57±0.53 % and 4.88±0.19 % at maximum in concentrations of from 8.33 to 100.00 μmol L^−1^ (Figure 4). As well, with three hydroxyl moieties bonding to molecular backbone of flavone, apigenin as a potential inhibitor in possession of three hydroxyl groups localized at 5,7,4’‐positions has a better ability of XOD inhibition, with the inhibitory effects of from 24.65±0.57 % to 32.81±1.93 % (Figure 4). Nevertheless, when ‐OH at 4’‐position of apigenin transferred to the 3‐ or 6‐ position, the inhibitions for galangin and baicalein are being on decline to varying degrees, so far as to being negative effect. Above experimental data implied well that the ‐OH localized at 4’‐position of flavone molecular backbone is appeared to be much more dominant in the inhibition of XOD, as comparing to ‐OH placed at 3‐ or 6‐position, while the 3‐OH of flavone backbone is seemly to inadequately contribute for inhibiting bio‐activity of XOD. Furthermore, with a similar structure of daidzein (7, 4’‐OH), the inhibitory performance of isoflavone genistein comprising hydroxyl moieties at 5, 7, 4’‐positions is close to that of daidzein, indicating that ‐OH at 5‐position of isoflavone framework may not seemly contribute for the inhibition of XOD. Four hydroxyl groups of fisetin, kaempferol and luteolin (LUT) on the whole can work well in inhibiting XOD bio‐activity, especially for luteolin bearing 5, 7, 4’, 5’‐OHs with the maximum inhibitory effect of 61.09±0.68 % and IC_50_ 7.87 μmol L^−1^ (Figure 4). Kaempferol (3, 5, 7, 4’‐OH) and fisetin (3, 7, 3’, 4’‐OH) containing dis‐ and advantaged functional groups of 3‐ and 4’‐OH are regarded as effective agents for inhibiting XOD activity, but their performances are less than the exhibition of luteolin on inhibitory effects, implying that ‐OH at 3‐position of flavone framework is indeed a disadvantaged functional group for inhibiting XOD activity, as well as the 3’‐OH attached to B ring of fisetin being more predominant than the 5‐OH at A ring of kaempferol. Flavonols possessing five hydroxyl moieties, such as quercetin (QUE) and morin are electron‐enriching systems. The differences between them are mainly the locations of hydroxyl groups on B ring. QUE and morin belong to planar molecules. When confronted XOD, QUE presents excellent performance in the effect of inhibiting XOD, with IC_50_ of 25.89 μmol L^−1^ (Figure 4). As well, morin is similar to QUE in molecular skeleton; the effect of inhibiting XOD is beyond our expectations with less performance, which should be attributed to the existence of 6’‐OH on the B ring. It convinced of us that the 6’‐OH on the B ring expresses negative response for inhibiting effect of XOD. Moreover, chrial flavonol of DMY (2R, 3R) originally isolated from medicinal vine plant of Ampelsis grossedentata or Vine tea, is the most abundant bioactive component of Vine tea, which was found to exhibit multiple biological effects including anti‐cancer, anti‐bacterial, anti‐inflammatory and anti‐microbial.^[17]^ Based on our previous works,[^18^, ^19^] DMY isolated in our laboratory had been evidently testified to be a good superoxide‐anion scavenger and strong reductant for synthesis of DMY‐metal complexes. DMY presenting one alkyl‐ and five phenolic hydroxyl moieties was investigated for evaluating its inhibiting effect of XOD. The IC_50_ value of 20.96 μmol L^−1^ in inhibition was pleasure to be a potential inhibitor for XOD activity. Myricetin (MY) is a dehydrogenation product of DMY, performing excellent inhibitory effect (IC_50_ 28.79 μmol L^−1^) and a good concentration‐dependent manner. It indicated that non‐planar molecular skeleton may be the better profit for the interaction of MY with XOD in a comparison with non‐planar DMY.

Hydroxyl usually acting as a hydrogen‐bonding donor can combine with residues of protein via H‐bonding interactions. Flavonoids containing multi‐hydroxyls perform good inhibitions for XOD activity. However, to further witness the role of hydroxyls on flavonoids played in the inhibition of XOD, natural flavone of nobiletin in possession of six methoxy groups can't work effectively in inhibiting XOD bio‐activity, although the resulting data is positive at certain range of concentrations. The cause is possibly ascribed to the facts that all of hydroxyl groups adhered to molecular skeleton are screened by methyl moieties, losing their capacities to responding for enzyme via H‐bonding interactions. Another natural product of jaceosidin with partial methoxy groups at 6, 3’‐positions is identified to be a potent agent, with 27.77±0.71 % in inhibitory effect(Figure 4). The resulting evidences further supported that ‐OH at 5, 7, 4’‐positions of flavone skeleton can effectively promote up the effect of inhibiting XOD activity.

Notably, based on above experimental data, we found that the inhibitory results of some bioflavonoids are in difference from those reported in the literature, which are shown in Table 1. MY acting as one of flavonols being on the IC_50_ value of 28.79 μmol L^−1^ in our work, is obviously much more than the IC_50_ values of 8.66, 0.24 μmol L^−1^ and 1.27, 2.38 μmol L^−1^ reported in the documents, which were assayed respectively by the test method A and B. As well, in the reported method A and B, these IC_50_ values of 2.74, 2.38 μmol L^−1^ and 0.44, 2.62 μmol L^−1^ on QUE inhibiting XOD activity are apparently much less than that of 25.89 μmol L^−1^ obtained from the improved test method. It implied that the chemical affinity of QUE with XOD in the improved test method (test method C) is weaker. Furthermore, although LUT is capable to be an effective tool for inhibiting XOD activity *in vitro*, with IC_50_ value of 7.87 μmol L^−1^, its IC_50_ value assayed by the test method C is still higher in data, indicating that its chemical affinity of XOD is not much better, as compared to the IC_50_ value of less than 1 μmol L^−1^ in the reports. Nevertheless, the IC_50_ value of 20.96 μmol L^−1^ on DMY determined by the test method C is less than that of 57.90 μmol L^−1^ in the report, implying that the potential of DMY on the inhibition of XOD is good. Emphatically, other flavonols, flavones and isoflavonoids, such as fisetin, galangin, kaempferol, morin, chrysin, apigenin, baicalein, daidzein and genistein, exhibit less than 50 % of inhibitory effects, their inhibitions are distinguished from that of them in the reports with good IC_50_ values. So, the difference on inhibitory effects of bioflavonoids assayed by above three test methods causes of us to think deeply.

**Table 1:** IC_50_ values of bioflavonoids afforded by varied test methods.

Species	Bioflavonoids	Methods^[a]^	IC_50_/*μ*mol L^−1^
Flavonols	Myricetin	Test method A	8.66^[8]^, 0.24^[22]^
		Test method B	1.27^[13]^, 2.38^[14]^
		Test method C	28.79
	Quercetin	Test method A	2.74^[9]^, 6.45^[20]^
		Test method B	0.44^[13]^, 2.62^[14]^
		Test method C	25.89
	Fisetin	Test method A	4.33^[14]^
		Test method B	140.00^[21]^
		Test method C	–
	Galangin	Test method A	163.00^[12]^
		Test method B	1.80^[14]^
		Test method C	–
	Kaempferol	Test method B	0.67^[13]^, 1.06^[14]^
		Test method C	–
	Morin	Test method B	10.10^[14]^
		Test method C	–
	Dihydromyricetin	Test method A	57.90^[17]^
		Test method C	20.96
Flavones	Chrysin	Test method A	1.26^[11]^
		Test method B	5.02^[13]^, 0.84^[14]^
		Test method C	–
	Lutelin	Test method A	0.21^[22]^
		Test method B	0.96^[13]^, 0.55^[14]^
		Test method C	7.87
	Apigenin	Test method B	0.70^[14]^
		Test method C	–
	Baicalein	Test method B	2.79^[14]^
		Test method C	–
Isoflavonoids	Daidzein	Test method B	>100^[13]^
		Test method C	–
	Genistein	Test method B	83.00^[13]^
		Test method C	–

Note: ^[a]^ the test method A is required for the mixture of XOD and inhibitor to be pre‐incubated, before the reaction is initiated by the addition of XAN; the test method B is required for XOD to be added to the mixture of XAN with inhibitor; the test method C is the improved test method in our work, with the generation of ⋅O_2_^−^.

### Comparison of Test Methods on Inhibiting XOD Activity in vitro

**Scheme 1:**
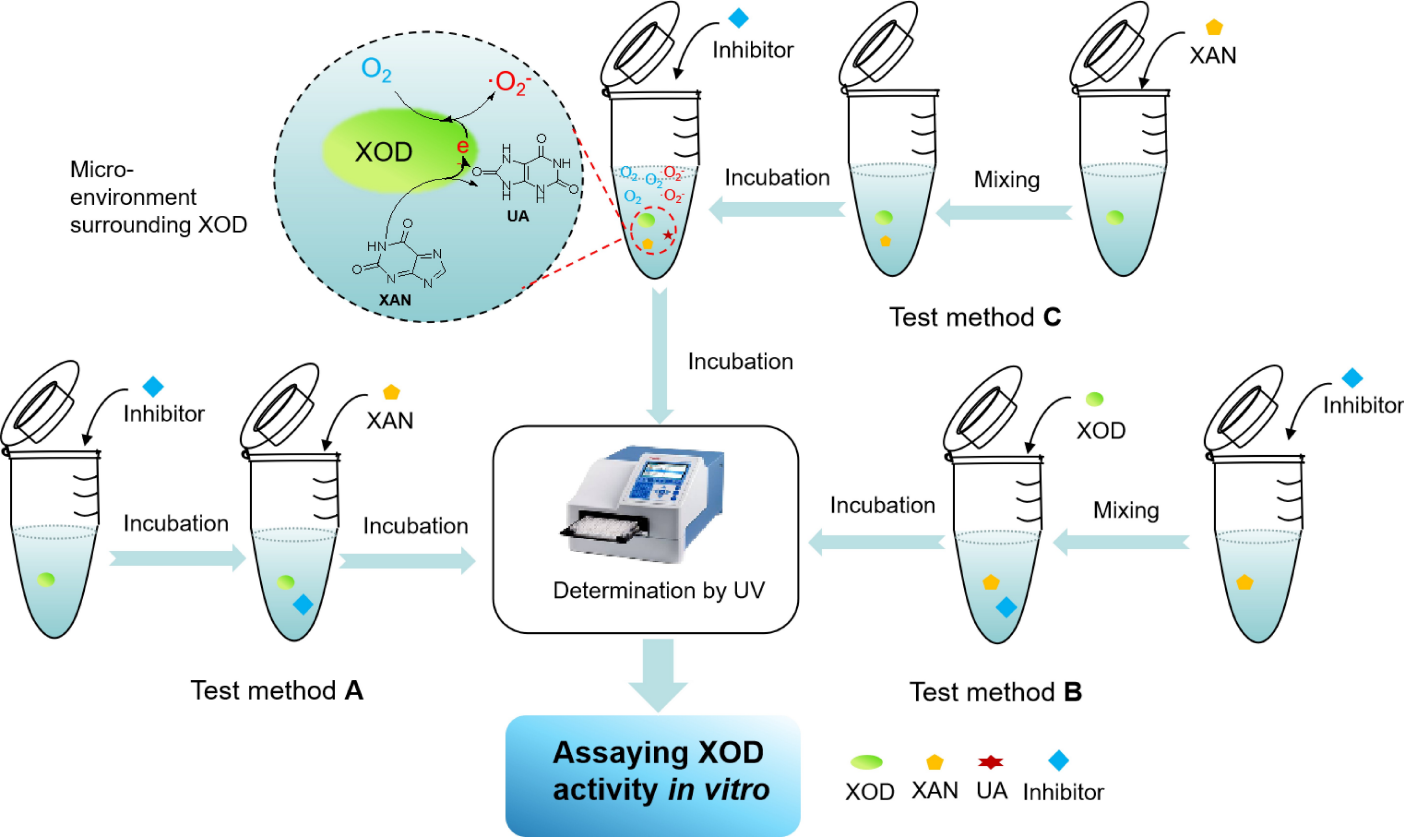
Introduction of three test methods applied to assaying XOD activity *in vitro*.

As described on Scheme 1, in the test method A, it required for XAN to initiate the oxidation, after the interaction between XOD and inhibitor occurred. As for the test method B, the reaction was permitted to be started by the addition of XOD into the mixture of XAN with inhibitor. Simultaneously, the test method C in this work asked for the oxidation by XOD to proceed for a while, then the inhibitor was permitted to be added for evaluating its inhibitory effect. At present, the test method A applied to assaying XOD inhibition *in vitro* has been much more popular, but is seemly different from the estimation on XOD inhibition *in vivo*. Because the micro‐environment surrounding XOD *in vivo* is a dynamic process with the generation of ⋅O_2_^−^. Afterwards, via the diagnosis for the three test methods, it found that the micro‐environment surrounding XOD *in vitro* in the test method C is the presence of ⋅O_2_^−^. In other words, potential inhibitor has to tolerate the attack of ⋅O_2_^−^generated from XOD, before the interaction of inhibitor with XOD occurred. Inversely, the test methods (A and B) are authorized to permit inhibitor to interact with XOD in the absence of ⋅O_2_^−^. Comprehensively, the explanation on the difference of inhibitory effects of bioflavonoids in three test methods could be elaborated by ⋅O_2_^−^ affecting molecular skeleton of bioflavonoids, resulting in H‐bonding interactions with XOD damaged partially.

**Figure 5:**
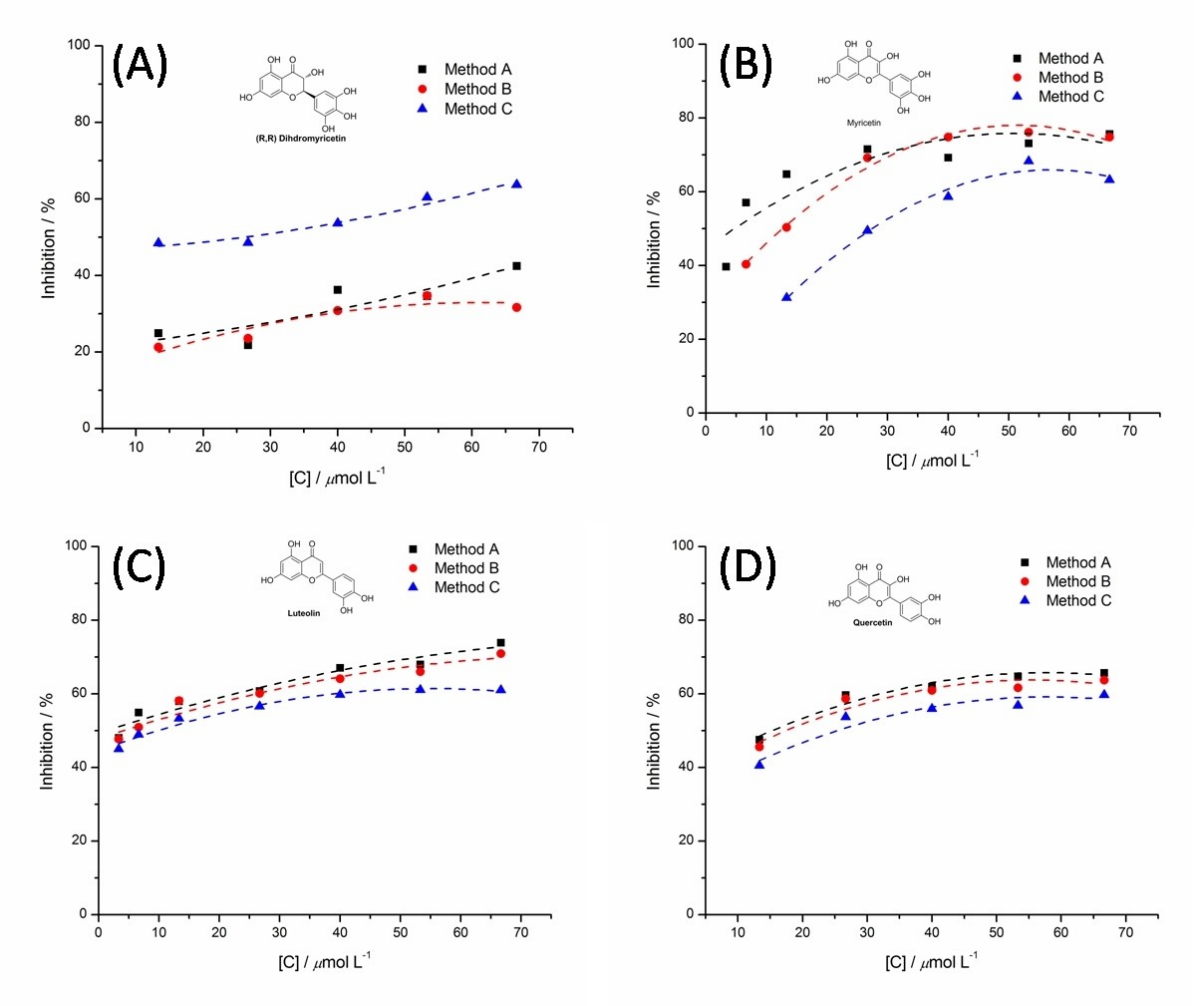
The inhibitions of DMY, MY, QUE and LUT on XOD activities by the test methods (A, B and C).

To witness further the proposition, MY, QUE, LUT and DMY exhibiting better inhibitions *in vitro* were selected to be promising agents for discussing their inhibitions in the varied test methods. As shown in Figure 5(C), LUT with four hydroxyl groups is able to be on an effective agent for inhibiting XOD bio‐activity, as a result of good inhibitory performance *in vitro* assayed by the test methods, the IC_50_ values are respective 4.34, 5.15 and 7.87 μmol L^−1^, which are shown in Table 2. QUE, as a powerful flavonol, shows similar inhibitory effect to LUT in regulation with a slight decrease on the inhibition in the test method C (Figure 5(D)). With the increase of hydroxyl moiety on the molecular backbone, the difference on MY inhibiting XOD activity is obviously appeared up, which is depicted in Figure 5(B). Compared with inhibitory results confirmed by the test methods (A and B), the inhibitory effects of MY determined by the test method C are significantly lower. However, DMY in possession of six hydroxyl moieties was investigated to evaluate its inhibition on XOD activity in varied test methods. As described in Figure 5 (A), it appeared well that the inhibitory effects of DMY perform to be a good concentration‐dependent manner at a certain range of concentrations. The IC_50_ value of 20.96 μmol L^−1^ determined by the test method C indicates a better chemical affinity for XOD, in comparison to the absence of IC_50_ values assayed by the test method A and B( Table 2).

**Table 2:** Comparison of three test methods on IC_50_ values.

Entry	Bioflavonoids	Range of concentrations /*μmol L* ^−1^	IC_50_^[a]^/*μ*mol L^−1^	IC_50_^[b]^/*μ*mol L^−1^	IC_50_^[c]^/*μ*mol L^−1^
1	Dihydromyricetin	13.33 −66.67	–	–	20.96
2	Myricetin	3.33–66.67	5.00	11.24	28.79
3	Quercetin	13.33–66.67	14.31	16.41	25.89
4	Luteolin	3.33–66.67	4.34	5.15	7.87

Note: ^[a]^ in the test method A; ^[b]^ in the test method A; ^[c]^ in the test method C.

**Scheme 2:**
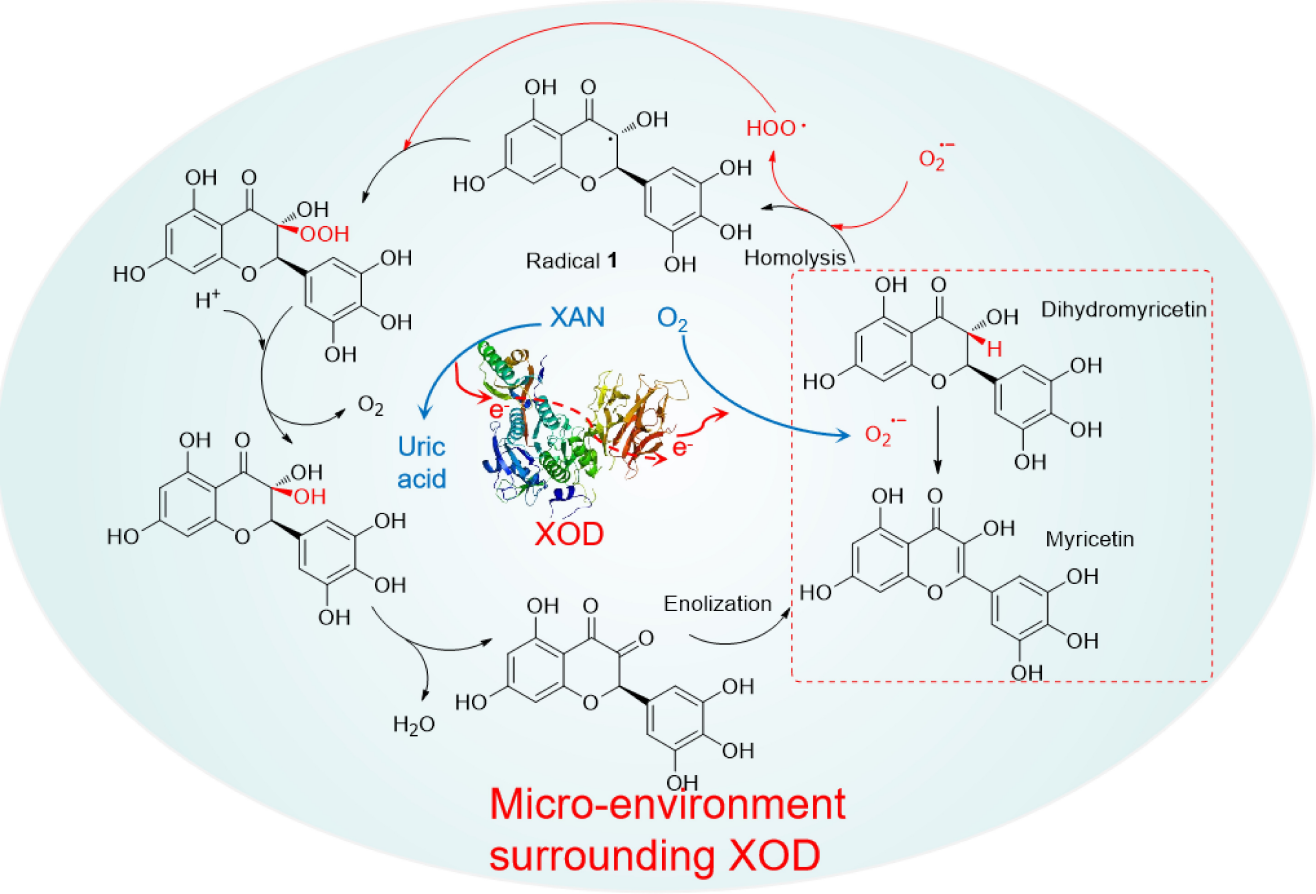
The rational pathway of DMY transformed into MY in the presence of ⋅O_2_^−[21]^

As far as we known, most of bioflavonoids are capable to be good superoixde‐scavengers. The process of bioflavonoids scavenging ⋅O_2_^−^ is actually a free radical redox reaction. The structures of bioflavonoids are vulnerable to be damaged to decline their bio‐activities, when confronts ⋅O_2_^−^. Of course, this is a possibility. However, there was a work documented in our laboratory that DMY is able to be selectively oxidized into MY in the presence of ⋅O_2_^−^, with good superoxide‐scavenging performance.^[18]^ The rational pathway involving in free radical oxidation had been proposed rationally. The pathway was illustrated on Scheme 2. A C−H localized at 3‐position of molecular backbone is firstly dissociated equally into corresponding radical **1**, with hydroperoxide anion (HOO⋅) produced, in the presence of ⋅O_2_^−^. Then the relative peroxide is permitted to be formed by the combination of radical **1** with peroxyl radical HOO⋅. Subsequently, the peroxide is flexibly decomposed with releasing O_2_ in the presence of H^+^. Finally, undergoing removing H_2_O and enolization, the product of MY could be afforded.^[18]^ Maybe this explanation on synergistic effect of MY can support the good inhibitory results of DMY on the inhibition of XOD, which was assayed by the test method C.

**Figure 6:**
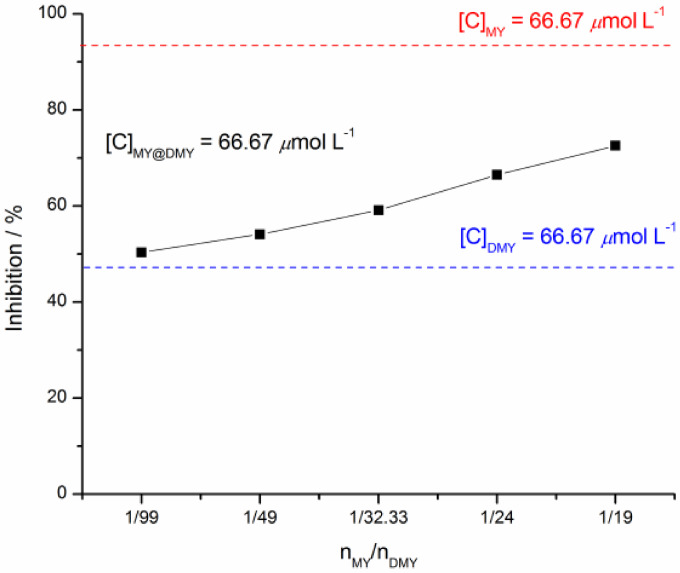
The mixture of DMY and MY in varying molar ratio inhibiting XOD activities *in vitro*.

What's more, to further testify the synergistic effect of MY on DMY inhibiting XOD activity, the trial was planned to investigate the inhibition of DMY on XOD in the presence of MY. Herein, it is a necessity of the test method A to be followed. The mixture of XOD with DMY and MY was prepared, then the reaction started by adding XAN substrate. Their inhibitory results demonstrated that with the molar ratio of n_MY_/n_DMY_ increasing from 0.67/66 to 3.33/63.33, their inhibitory effects are obviously better excellent than that of pure DMY at a concentration of 66.67 μmol L^−1^, which is asked to be a control group (Figure 6). It indicated that MY can contribute for the inhibition of DMY on XOD, performing a good synergistic effect.

## Conclusions

The concentration on bioflavonoids inhibiting XOD activity has been paid much more by bio‐chemists all the time. Excellent inhibitions of bioflavonoids on XOD activity *in vitro* have been certificated to be an accessible gateway to the treatment of hyperuricemia. Therefore, developing a potent approach to exploring potential inhibitor is much significant. In this work, the test method for assaying XOD activity *in vitro* has been improved to be a strong tool, in which there is a micro‐environment surrounding XOD created in the presence of ⋅O_2_^−^.The improved test method asks for potential inhibitor to tolerate the ⋅O_2_^−^attack,before the interaction of XOD with inhibitor occurred, that is essentially different from test methods reported in the literature. In addition, there are some bioflavonoids to be investigated by the improved test method. Bioflavonoids of DMY, MY, QUE and LUT have been testified to be potent inhibitors of XOD, with IC_50_ values of 20.96, 28.79, 25.89 and 7.87 μmol L^−1^, although these IC_50_ values are in difference from them reported in the documents. All in all, the improved test method for assaying XOD activity is witnessed to be an effective gateway to hunting for potential inhibitors *in vitro*, resulting from the micro‐environment surrounding XOD created in the improved test method being consistent with that produced *in vivo*, with the generation of ⋅O_2_^−^.

## Author Contributions

Yuanyong Yao and Tao Wu were in charge of designing the experiments. And they were regarded as co‐first author with equal contribution for writing manuscript. Yuanyong Yao, Yan Zhang, Daihua Fu and Meng Zhang performed experiments. All authors contributed to the article and approved the submitted version.

## Conflict of Interests

The authors declare no conflict of interest.

1

## Supporting information

As a service to our authors and readers, this journal provides supporting information supplied by the authors. Such materials are peer reviewed and may be re‐organized for online delivery, but are not copy‐edited or typeset. Technical support issues arising from supporting information (other than missing files) should be addressed to the authors.

Supporting Information

## Data Availability

The data that support the findings of this study are openly available in Supporting Information at https://www.[url], reference number 1.
