# Supplementary material for: An Improved Test Method for Assaying the Inhibition of Bioflavonoids on Xanthine Oxidase Activity in vitro
Source: ChemistryOpen. 2024 Sep 9;13(12):e202400127. doi: 10.1002/open.202400127 (PMC11625940; doi:10.1002/open.202400127)
Supplement: Supplementary file 1 — Supporting Information [file OPEN-13-e202400127-s001.pdf]

ChemistryOpen

Supporting Information

An Improved Test Method for Assaying the Inhibition of Bioflavonoids on Xanthine Oxidase Activity *in vitro*

Yuanyong Yao,* Tao Wu, Meng Zhang, Daihua Fu, Hai Yang, and Shixue Chen*

Supplementary Material

An improved test method for assaying the inhibition of bioflavonoids on xanthine oxidase activity *in vitro*

Yuanyong Yao * [a][b], Tao Wu[b], Meng Zhang[b], Daihua Fu[b][c], Hai Yang[b], Shixue Chen[b] *

[a] State Ethnic Affairs Commission Key Development Laboratory of Chinese Veterinary Medicine & National and Local Joint Engineering Center of Chinese Veterinary Medicine Separation and Purification Technology, Tongren Vocational and Technical University, Tongren 554300 (China)

[b] Institute of Material and Chemical Engineering, Tongren University, Tongren 554300 (China)

[c] Key Laboratory of Medicinal Chemistry for Natural Resource of Ministry of Education and Yunnan University, Kunming 650091 (China)

** Correspondence:*

Yuanyong Yao

E-mail: chyyyy@gztrc.edu.cn (Y. Y.);

Shixue Chen

E-mail: Tongrencsx01@126.com (C. S).

Keywords: Test method; Xanthine oxidase; Bioflavonoids; In vitro inhibition

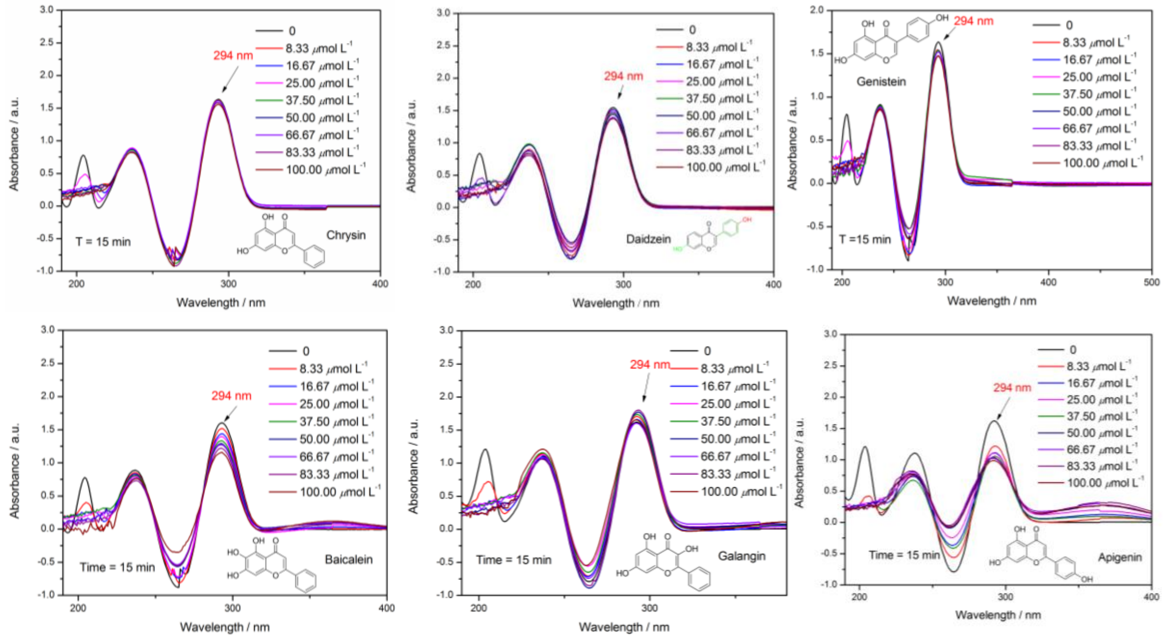

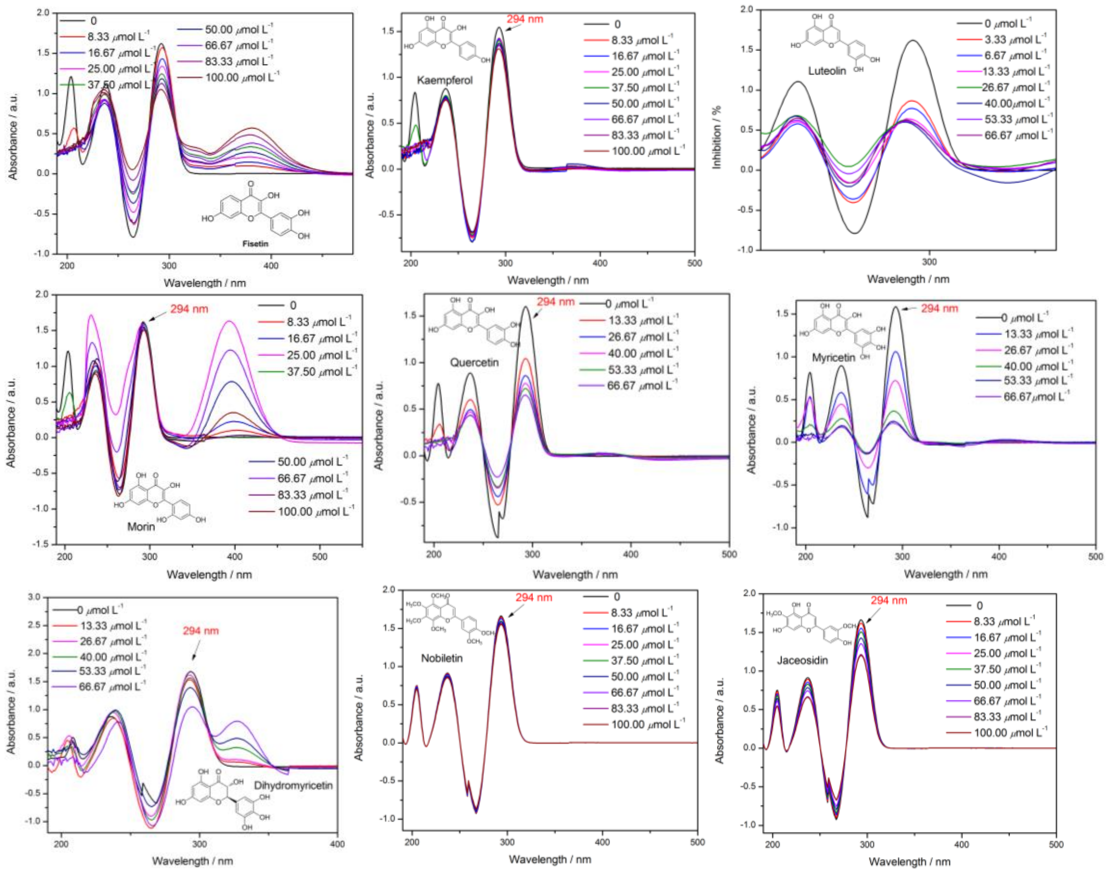

Fig S1 UV spectrums of bioflavonoids inhibiting XOD in vitro
